# Long-term exposure to PM_2.5_ air pollution and mental health: a retrospective cohort study in Ireland

**DOI:** 10.1186/s12940-024-01093-z

**Published:** 2024-06-10

**Authors:** Seán Lyons, Anne Nolan, Philip Carthy, Míde Griffin, Brian O’Connell

**Affiliations:** 1 School of Economics, University College Dublin, Dublin, Ireland; 2 Department of Economics, Trinity College Dublin, Dublin, Ireland; 3 Economic and Social Research Institute, Whitaker Square, Sir John Rogerson’s Quay, Dublin 2, D02 K138 Ireland; 4 The Irish Longitudinal Study on Ageing, Trinity College Dublin, Dublin, Ireland; 5 Dublin Dental University Hospital, Trinity College Dublin, Dublin, Ireland

**Keywords:** Environmental health, PM_2.5_, Particulate air pollution, Mental health, Depression, Anxiety, Ireland

## Abstract

**Background:**

Mental illness is the leading cause of years lived with disability, and the global disease burden of mental ill-health has increased substantially in the last number of decades. There is now increasing evidence that environmental conditions, and in particular poor air quality, may be associated with mental health and wellbeing.

**Methods:**

This cross-sectional analysis uses data on mental health and wellbeing from The Irish Longitudinal Study on Ageing (TILDA), a nationally representative survey of the population aged 50+ in Ireland. Annual average PM_2.5_ concentrations at respondents’ residential addresses over the period 1998–2014 are used to measure long-term exposure to ambient PM_2.5_.

**Results:**

We find evidence of associations between long-term exposure to ambient PM_2.5_ and depression and anxiety. The measured associations are strong, and are comparable with effect sizes for variables such as sex. Effects are also evident at relatively low concentrations by international standards. However, we find no evidence of associations between long-term ambient particulate pollution and other indicators of mental health and well-being such as stress, worry and quality of life.

**Conclusions:**

The measured associations are strong, particularly considering the relatively low PM_2.5_ concentrations prevailing in Ireland compared to many other countries. While it is estimated that over 90 per cent of the world’s population lives in areas with annual mean PM_2.5_ concentrations greater than 10 μg/m^3^, these results contribute to the increasing evidence that suggests that harmful effects can be detected at even low levels of air pollution.

## Introduction

The World Health Organization (WHO) estimates that 4 million premature deaths every year are a result of ambient (outdoor) air pollution [1]. The burden of disease attributable to air pollution is now estimated to be comparable with other major global health risks such as unhealthy diet and tobacco smoking, and was in the top five out of 87 risk factors for male and female deaths in 2019 [2]. As a result, air pollution is now recognised as the single largest environmental threat to public health [1]. Although air pollution has decreased in most European countries over the past two decades, including Ireland, levels of ambient air pollution remain above WHO guidelines in many cities and towns in Ireland [3].

Air pollution contains many individual pollutants, including particulate matter (PM), gaseous pollutants and metallic and organic compounds [4]. While the European Union and international organisations such as the WHO issue guidelines in relation to multiple types of air pollution, exposure to fine particulate matter of 2.5 microns or less in diameter (PM_2.5_) is considered to be particularly damaging to health [1, 2]. PM_2.5_ particles can penetrate and lodge deep inside the lungs, and, along with ultrafine particles, may even enter the blood system affecting major organs [1]. There is now strong causal evidence of associations between exposure to PM_2.5_ and all-cause mortality, as well as acute lower respiratory infections, chronic obstructive pulmonary disease (COPD), ischaemic heart disease (IHD), lung cancer and stroke [1, 5–7]. Recent systematic reviews have also shown strong evidence of associations between PM_2.5_ and other health outcomes such as diabetes [8], infant health [9], cognitive functioning [10] and dementia [11]. The available evidence also suggests that the health-damaging effects of PM_2.5_ air pollution operate even at low exposure levels [1, 5]. As a result, the new WHO air quality guidelines (AQGs) are now set at levels of 5 μg/m^3^ (annual mean) and 15 μg/m^3^ (24-h mean), levels substantially below those that were in place before 2021 [1].

While the bulk of past research focuses on the effects of PM_2.5_ on physical health and mortality, some recent research has also found evidence of associations between exposure to ambient PM_2.5_ air pollution and mental health and wellbeing. For example, a recent systematic review of 22 studies supports the hypothesis that there could be an association between long-term PM_2.5_ exposure (> = 6 months) and mental health outcomes including depression and anxiety [4]. For depression prevalence, they report a pooled odds ratio of 1.102 per 10-μg/m^3^ increase in PM_2.5_ exposure (95% CI: 1.023, 1.189) (based on a meta-analysis of five studies). In addition, two of the included studies found statistically significant positive associations between long-term PM_2.5_ exposure and the prevalence of anxiety symptoms above a threshold that was considered clinically relevant. Another meta-analysis of six cohort and two cross-sectional studies found a statistically significant relationship between long-term (> = 1 year) exposure to PM_2.5_ (OR = 1.06, 95% CI: 1.00, 1.13 per 5 μg/m^3^ increase) and depression prevalence [12]. Other studies using a variety of statistical methodologies, as well as indicators for air pollution exposure, are suggestive of an association between ambient air pollution and other indicators of mental health and wellbeing such as the incidence of schizophrenia spectrum disorder [13], and the prevalence of suicide [14], anxiety [13, 15], depression [13], bipolar disorder [16] and life satisfaction [17].

Despite this growing evidence base on the links between ambient air pollution and mental health, most of the previous studies used data on relatively short-term exposures. For example, the systematic review by [4] identified 22 qualifying studies on this topic, but only five of these examined long-term exposures (defined as over six months). However, some hypothesised channels through which PM_2.5_ might affect mental health are likely to operate over a longer exposure period [4, 12, 16], including:

- Inflammation affecting the central nervous system;
- Changes in stress responsivity, via hypothalamic-pituitary-adrenal (HPA) axis activation; and
- Adverse effects on cognitive development and dementia risk.

Focusing exclusively on short-term pollution exposures might miss or understate the impact of longer-term processes. In the present study, we have access to long-term residential histories for a large representative sample of people aged over 50 in Ireland. This cross-sectional analysis uses data on mental health and wellbeing from The Irish Longitudinal Study on Ageing (TILDA), a nationally representative survey of the population aged 50+ in Ireland. Annual average PM_2.5_ concentrations at respondents’ residential addresses over the period 1998–2014 are used to measure long-term exposure to ambient PM_2.5_.Since the available data are at individual level, we can also allow for a wide range of possible confounding socioeconomic factors.

## Data and methods

### Study population

The Irish Longitudinal Study on Ageing (TILDA) is a population-based, nationally-representative, longitudinal study of 8,504 community-dwelling adults in Ireland aged 50 and older and their partners of any age. The dataset contains a rich set of variables on the health and socio-economic circumstances of older people. The study is harmonised with other international longitudinal studies of ageing, such as the Survey of Health, Ageing and Retirement in Europe (SHARE), the English Longitudinal Study on Ageing (ELSA) and the Health and Retirement Study (HRS) in the US. Baseline data collection took place in 2009–2011, and participants have been followed up at two-year intervals since then. Data are collected via a variety of modes, including computer-aided personal interviewing (CAPI), a self-completion questionnaire (SCQ) and a nurse-led health assessment (the latter carried out in Waves 1, 3 and 6) [18, 19]. Data from the CAPI and SCQ are used in this paper.

**Fig. 1:**
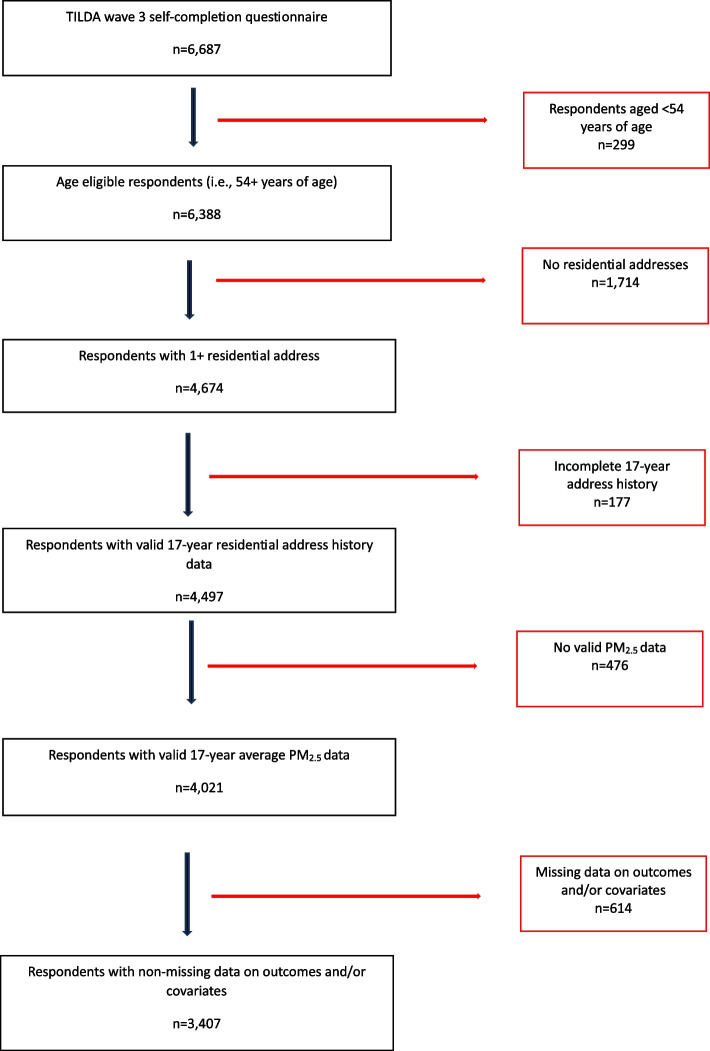
Flowchart of study selection criteria

In this study, we use data from the self-completion questionnaire (SCQ) in Wave 3 (collected in 2014/2015) as the Wave 3 SCQ included a module on residential address history (*n* = 6,687). The questionnaire provided space for respondents to provide exact address details for up to ten locations where they have resided, starting with the most recent. A geocoded dataset of the responses to this questionnaire, supplemented with the current recorded addresses of study participants as collected as part of the primary TILDA interview, was provided to the researchers for the present study (see also Appendix 1). After deletion of those who were not age-eligible (i.e., aged less than 54 years of age) (*n* = 299) and those who did not complete the residential address history questionnaire (*n* = 1,714), 4,674 observations remained for matching with PM_2.5_ data. As the data on ambient air pollution are available only from 1998 (see Data on ambient air pollution section), we select those respondents with a complete address history for each of the 17 years 1998–2014 inclusive, and assign the PM_2.5_ concentration in their area in the relevant year to their address in that year (*n* = 4,021). Deletion of respondents with missing data on outcomes and/or covariates results in a final sample size of 3,407 respondents. Figure 1 details the study selection criteria in further detail.

### Data on ambient air pollution

We use data on global estimates of annual average PM_2.5_ concentrations at 0.01 degree resolution (approximately a 1km grid) between 1998 and 2014 to characterise ambient air pollution at TILDA respondents’ addresses. These data are downloaded from [20] and described in [21]. In essence, satellite sensors measure particulates blocking various wavelengths of light in a column of air. The concentration of PM_2.5_ air pollution in each grid cell across the world is modelled by calibrating the sensor readings to reflect direct ground-based estimates in places where measurements are available and applying a chemical transport model of the atmosphere.

The main explanatory variable of interest (${E}_{i}$) is therefore a proxy for long-run exposure to ambient air pollution. It was calculated by taking the arithmetic mean of the annual PM_2.5_ concentrations (${C}_{it}$) in the 1 km grid square in which each TILDA respondent resided in each year for the 17 years prior to the collection of outcomes data (1998–2014):

1 $$\begin{array}{c}{E}_{i}=\frac{\sum_{t=1998}^{2014}{C}_{it}}{17}\end{array}$$

To protect respondents’ confidentiality, we rounded the long-run concentration to the nearest 1 μg/m^3^. The frequency distribution for this variable is shown in Table 1, with over half of the sample experiencing relatively low levels of exposure (5–7 μg/m^3^). The sample mean value of the rounded variable is 7.67 and the standard deviation is 1.54.

**Table 1:** Descriptive statistics for categorical PM_2.5_ exposure variable, rounded to nearest 1 μg/m^3^

**Rounded PM**_**2.5**_ **exposure (μg/m**^**3**^**)**	**Freq.**	**Percent**
5	102	2.99
6	663	19.46
7	1,043	30.61
8	888	26.06
9	179	5.25
10	255	7.48
11	251	7.37
12	26	0.76
	3,407	100

As indicated in Table 1, by far the most prevalent category is 7. We use this as the reference category in models with a categorical representation of PM_2.5_ concentrations.

**Fig. 2:**
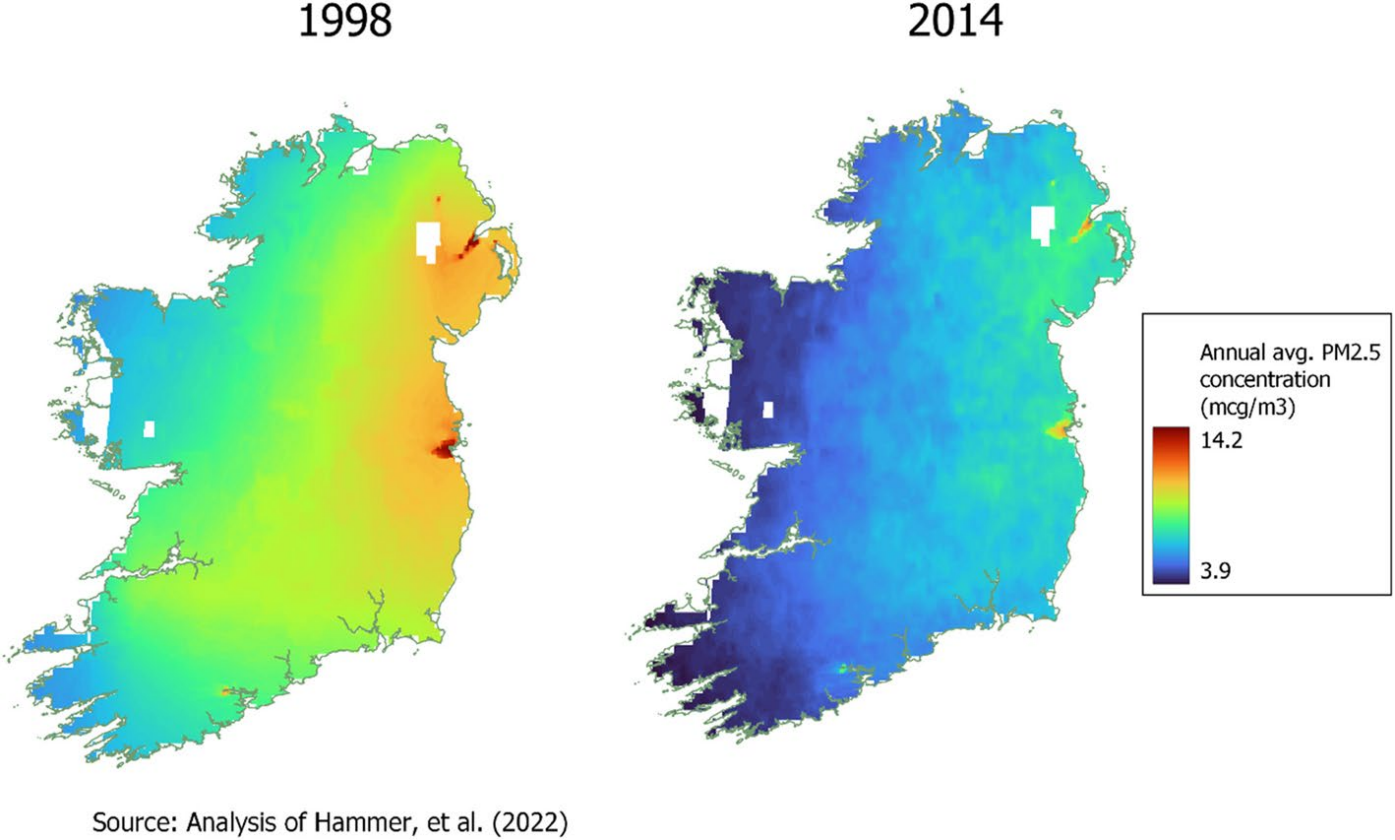
Annual average PM_2.5_ concentration in Ireland, 1998 and 2014, 0.01 degree resolution

To illustrate the main spatial features of our PM_2.5_ sample, Fig. 2 shows the annual maps for the start and end of the sample period. Concentrations generally fell over time. They also tended to be higher in eastern areas, particularly in the capital city, Dublin. This is consistent with the higher density of population and economic activity in the east, and the prevailing wind blows from the west. Some areas along the west coast, mainly in counties Mayo and Galway, were not included on the PM_2.5_ maps and thus some TILDA respondents are omitted from the analysis due to unavailability of pollution data for them.

### Data on mental health outcomes

Five indicators of mental health and wellbeing are examined in this study. Scores from the 8-item Center for Epidemiologic Studies Depression Scale (CES-D) are used to measure depression [22]. This validated measure captures the frequency with which respondents report experiencing a series of depressive symptoms within the past week. The items included consist of statements such as ‘I felt depressed’, ‘I felt that everything I did was an effort’, ‘my sleep was restless’, ‘I enjoyed life’. These statements are presented to the respondents during the CAPI and the respondents indicate how often they experienced these feelings (rarely or none of the time (less than 1 day), some or a little of the time (1–2 days), occasionally or a moderate amount of time (3–4 days), or all of the time (5–7 days)). The total number of positive and negative responses to each item are summed to obtain an overall score (answers to positive statements are reverse coded). Higher scores indicate increased depressive symptomology.

Anxiety is measured using the 7-item Anxiety subscale of the Hospital Anxiety and Depression scale (HADS-A), administered to respondents during the CAPI [23]. Items include ‘I felt tense or wound up’, ‘Worrying thoughts go through my mind’ and respondents are asked to indicate how often they felt this way during the past week (‘most of the time’, ‘a lot of the time’, ‘from time to time, occasionally’, ‘not at all’). As with the CES-D, the total number of positive and negative responses to each item are summed to obtain an overall score (answers to positive statements are reverse coded), with higher scores indicating increased anxiety.

The 8-item Penn State Worry Questionnaire is included in the SCQ [24]. The items include statements such as ‘my worries overwhelm me’, ‘many situations make me worry’, ‘I know I should not worry about things, but I just cannot help it’. The respondents are asked to indicate how typical or characteristic each statement is on a five-point scale, from 1 ‘not at all typical’ to 5 ‘very typical’. Responses to each item are then summed to obtain a total score, ranging from 8 to 40 (with some evidence of ‘bunching’ at even scores; see Fig. 2). Higher scores indicate a higher level of worry.

The 4-item version of the Perceived Stress Scale (PSS) is used to record stress in the SCQ. The PSS consists of four questions that asks respondents to indicate how they felt in the past month, with answers on a 5-point Likert scale from 0 (Never) to 4 (Very often). A sample item is ‘how often have you felt that you were unable to control the important things in your life?’. The range of the PSS score is [0,16], with a higher score indicating higher levels of perceived stress. Although the 4-item version of the PSS asks about how the respondents felt in the past month, it can be used as a measure of chronic stress associated with generalised stress perception and can reflect how unpredictable, uncontrollable, and overloaded an individual’s life is [25].

**Fig. 3:**
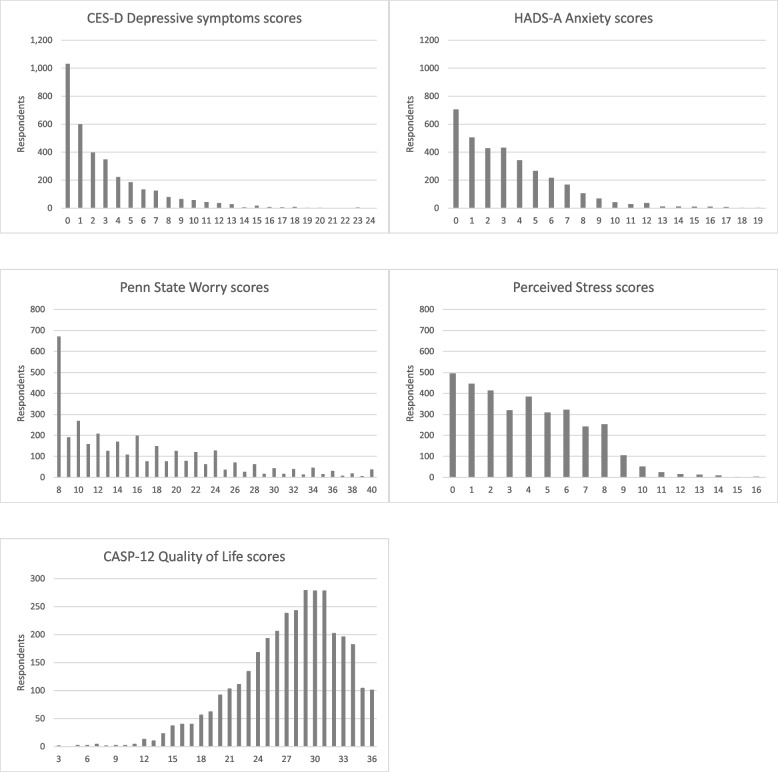
Sample frequency distributions for mental health and well-being indicators (*n* = 3,407)

Quality of life is an important measure of overall wellbeing and it is measured using the 12-item Control-Autonomy-Self-Realisation-Pleasure (CASP) scale covering four domains: control (the ability to actively participate in one’s environment), autonomy (the right of the individual to be free from unwanted interference of others), self-realisation (the fulfilment of one’s potential) and pleasure (the sense of happiness or enjoyment derived from engaging with life). The items included in those domains consist of statements such as: ‘I look forward to each day’, ‘my health stops me from doing the things I want to do’, ‘I feel that life is full of opportunities’. These statements are presented to the respondents in the SCQ and they are asked to indicate how often (often, sometimes, not often or never) they feel each statement applies to their life. The overall score is obtained by summing each item and higher scores denote better quality of life (answers to the negative statements are reverse coded) [26]. Figure 3 illustrates the frequency distributions for each of the five outcome variables considered in this study.

### Covariates

A variety of individual-level covariates are included in the statistical models to take account of potentially confounding socio-demographic, socio-economic, health and behavioural characteristics. Tables 2 and 3 present summary statistics for each independent variable. In addition to controls for age, sex, marital status and socioeconomic status (proxied by employment status and highest level of education completed), controls are added for health status and health behaviours. As there is no universal access to public healthcare in Ireland, an indicator for medical card status (which grants those on low incomes access to free public healthcare) is also added to further proxy for health need.

**Table 2:** Descriptive statistics for continuous explanatory variables

**Variable**	**Units**	**Mean**	**Std. Dev.**	**Min**	**Max**
Age	years	66.3	8.41	54	95

**Table 3:** Descriptive statistics for categorical sociodemographic variables

**Variable**	**Category**	**N**	**%**
Sex	Male	1,587	46.58
	Female	1,820	53.42
Employment status	Employed	1,140	33.46
	Retired	1,622	47.61
	Other	645	18.93
Highest level of education	Primary/none	718	21.07
	Secondary	1,432	42.03
	Third/higher	1,257	36.89
Marital status	Not married	888	26.06
	Married	2,519	73.94
Long-term health limitation	No	2,672	78.43
	Yes	735	21.57
Alcohol consumption problem	No alcohol consumption problem	3,020	88.64
	Alcohol consumption problem	387	11.36
	Missing alcohol consumption	100	2.94
Polypharmacy	Not taking 5 or more medications	2,619	76.87
	5 or more medications	788	23.13
Medical card (free public health services)	No medical card	1,971	57.85
	Medical card	1,436	42.15
Smoking status	Past smoker	1,574	48.20
	Current smoker	1,482	43.50
	Never smoker	351	10.30
Total		3,407	100

### Statistical methods

As described above, the PM_2.5_ data are matched to residential address history data from TILDA, which in combination with detailed data from TILDA on a variety of mental health outcomes and important confounders, allows for the specification of regression models that estimate the association between long-term exposure to PM_2.5_ air pollution and mental health.

For each of the outcome variables, two variations of the variables are modelled: ordinal scales and threshold-based metrics. To assist comparability, each of the metrics based on an ordinal scale is Z-standardised. This involves dividing each score’s deviation from the sample mean by the sample standard deviation. The scales transformed in this manner are the CES-D Depressive symptoms scale, the HADS-A Anxiety scale, the Penn State Worry scale, the Perceived Stress scale and the CASP Quality of Life scale. Details of these scales are given in Data on mental health outcomes section. Coefficients in the Z-standardised models show how many standard deviations difference in the dependent variable are associated with a unit change in a given explanatory variable. Equation 2 illustrates the model, with the Z-standardised outcome for individual *i* in 2014–2015 $\left({H}_{i}\right)$ explained as a linear function of a constant, an estimate of the individual’s long-term exposure to ambient pollution for the previous 17 years (${E}_{i}$, discussed in Data on ambient air pollution section), a vector of socioeconomic controls ${\overline{{\text{X} }}_{i}}$ (see Covariates section) and a random error term ${\varepsilon }_{i}$.

2 $$\begin{array}{c}{H}_{i}=\alpha +{\beta }_{1}{E}_{i}+\overline{\upgamma }{\overline{\text{X}} }_{i}+{\varepsilon }_{i}\end{array}$$

As an alternative to the linear specification of pollution effects, we also estimate models with a categorical representation of annual PM_2.5_ concentrations (see Table 1). In these specifications, dummy variables are included to indicate observations with rounded average exposures at each step of 1 μg/m^3^, with 7 μg/m^3^ regarded as the reference category.

In some cases, being in the upper tail of a scale’s distribution can provide more clinically relevant information. Metrics in this category aim to detect risk of depression (CES-D score > = 9), Anxiety (HADS-A score > = 11) and the Penn Generalized Anxiety Disorder indicator (Worry scale > = 22). These threshold-based measures are modelled as binary (1/0) variables using logit regression. We express the results from these regressions as odds ratios.

## Results

Table 4 summarises the standardised coefficients and confidence intervals for the five indicators modelled in this paper. The scales for depressive symptoms and anxiety show strong positive associations with long-term average residential PM_2.5_ concentrations, with *p*-values of less than 1 per cent. There is little evidence that any of the other indicators (worry, stress or quality of life) are associated with particulate pollution levels. Full regression results for the models of depressive symptoms and anxiety are in Tables 6 and 7 in Appendix 2. As shown in these tables, estimated PM_2.5_ effects do not differ substantially between univariate models and versions that adjust for a range of potential confounding factors.

**Table 4:** Summary of linear coefficients on PM_2.5_ exposure (μg/m^3^) in models of Z-standardised mental health scales with full set of controls

**Outcome**	**Coef**	**95% confidence**		***P* value**
		**Low bound**	**High bound**	
Depressive symptoms scale (CES-D)	0.0312	0.0093	0.0531	0.0053
Anxiety scale (HADS-A)	0.0380	0.0158	0.0602	0.0008
Penn State Worry Questionnaire Scale	-0.0145	-0.0363	0.0073	0.1910
Perceived Stress Scale	-0.0000	-0.0225	0.0224	0.9960
CASP Quality of Life Scale	0.0143	-0.0076	0.0362	0.2020

**Fig. 4:**
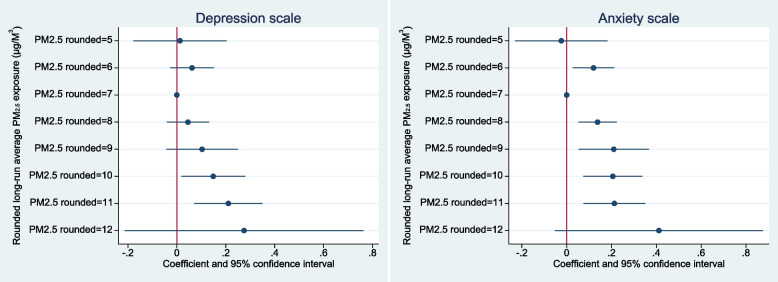
Coefficients on rounded PM_2.5_ exposure categories in fully adjusted OLS models of CES-D depressive symptoms Z-score and HADS-A anxiety Z-score; reference category = 7 μg/m^3^

To further explore the relationships between PM_2.5_ and the depressive symptoms and anxiety indicators, we re-estimate the models using a categorical representation of PM_2.5_ rather than assuming linearity. The pollution coefficients are illustrated for both models in Fig. 4, and the full regression results are included as Tables 8 and 9 in Appendix 2.

These figures reinforce the impression that higher PM_2.5_ exposures are associated with higher risk of depressive symptoms and anxiety. Indeed, in the case of depressive symptoms the relationship appears strikingly linear, at least above the reference category of 7 μg/m^3^. Information criterion tests tend to favour the linear PM_2.5_ specification over the categorical PM_2.5_ specification; for example, the linear depressive symptoms model has an AIC of 9,301 and a BIC of 9,393 compared to an AIC of 9,307 and a BIC of 9,436 for the categorical version. In Table 5, we investigate whether the results (with a linear specification of PM_2.5_) hold when the outcome variables for depression, anxiety and stress are expressed using threshold values. While there is now no evidence that long-term PM_2.5_ concentrations are associated with the binary indicator of clinically-significant depressive symptoms, the *p*-value for anxiety suggests an effect of PM_2.5_ on clinically-significant anxiety.

**Table 5:** Summary of odds ratios on PM_2.5_ exposure (μg/m^3^) in models of binary mental health metrics with full set of controls

**Outcome**	**Odds ratio**	**95% confidence interval**		***P* value**
		**Low bound**	**High bound**	
Depression threshold (CES-D score > = 9)	1.0657	0.9830	1.1550	0.1220
Anxiety threshold (HADS-A score > = 11)	1.2170	1.0850	1.3640	0.0008
Penn Worry scale threshold (PSS scale > = 22)	0.9750	0.9210	1.0320	0.3770

## Discussion and conclusions

Mental illness is the leading cause of years lived with disability, and the global disease burden of mental ill-health has increased substantially in the last number of decades [27]. There is now increasing evidence that environmental conditions, and in particular poor air quality, may be associated with mental health and wellbeing. We find evidence of associations between long-term exposure to ambient PM_2.5_ and validated indicators of depressive symptoms and anxiety for a large sample of over-50s in Ireland. Allowing for a range of potential confounding factors (age, sex, employment status, marital status, long-term health limitations, alcohol consumption problems, smoking status, polypharmacy and entitlement to free public healthcare) does not substantially affect these findings.

The measured associations are strong, particularly considering the relatively low PM_2.5_ concentrations prevailing in Ireland compared to many other countries. While it is estimated that over 90 per cent of the world’s population lives in areas with annual mean PM_2.5_ concentrations greater than 10 μg/m^3^ [21], these results contribute to the increasing evidence that suggests that harmful effects can be detected at even low levels of air pollution. To illustrate the strength of these relationships in our sample, note that moving from the reference category to the highest average PM_2.5_ exposure in our sample (7 to 12 μg/m^3^) involves an increase of 5 μg/m^3^. Multiplying the PM_2.5_ coefficient in the depressive symptoms model by 5 implies an increase of 16.2% of a standard deviation on the CES-D scale. This scale of effect is broadly comparable to the higher depressive symptom score among females (16.9%) compared to males and it is larger than the marginal effect of being in the subsample taking 5+ medications (13.6%), as shown in the full regression results for the depressive symptoms model (Table 6 in Appendix 2).

We find no evidence of associations between long-term ambient particulate pollution and other indicators of mental health and well-being: stress, worry and quality of life. Understanding why long-term PM_2.5_ concentrations are associated with depression and anxiety, but not other indicators of mental health and wellbeing, is challenging and worthy of further research. It is possible that different dimensions of mental health may be more or less influenced by the length of exposure, the specific type of pollutant and/or omitted confounding variables. We are aware of just one study [15] that investigate the impact of differing lengths of exposure to PM_2.5_ (and PM_10_ pollution) on mental health; using data from the US Nurses’ Health Study (age range 57–87), they found that exposure to fine particulate matter (PM_2.5_) was associated with higher symptoms of anxiety, with more recent exposures potentially more relevant than more distant exposures. However, these results are not directly comparable with the results in this study given the substantial difference in the study populations of interest.

While we have been able to control for many potential confounding factors at individual level, this is a cross-sectional study so it is not possible to draw conclusions about causality. Ideally, repeated measurements of mental health would be available for the 17-year period for which we have PM_2.5_ concentration data; in the absence of such data, this study adopted a cross-sectional approach investigating the link between 17-year annual average PM_2.5_ concentrations and mental health and wellbeing, measured in 2014/2015. In addition, pollution exposures were not randomly assigned to respondents, so there may have been some selection away from polluted areas among those able to afford better environments or those particularly affected by air pollution. Future work could exploit ‘natural experiments’, such as policy changes, to identify the causal impacts of air pollution on mental health. See [27] for an application using data from the China Health and Retirement Study (CHARLS), a sister study of TILDA.

Measured effects may also have been influenced by omission of potentially important correlated factors such as other air pollutants or noise exposure [15]. The WHO note that in everyday life, individuals are exposed to a mixture of air pollutants that varies in space and time [1]. It is therefore possible that the association we observed for long-term PM_2.5_ is attributable, in whole or in part, to a correlation between PM_2.5_ and another exposure. For example, [16] find a large and statistically significant positive association between average annual ambient local PM_2.5_ concentrations in 2010 and a broad indicator of depression based on self-reported symptoms of nerves, anxiety, tension or depression (using data on adults aged 40–69 from the UK Biobank). The odds of reporting one or more of these symptoms is reported to be 2.31 (95% CI: 2.15–2.50) times higher per 10 μg/m^3^ increase in PM_2.5_. Positive associations are also reported for indicators of major depression and bipolar disorder. The authors also find an independent association between mental health outcomes and a modelled proxy for road traffic noise, highlighting the difficulty in assessing the independent effects of different pollutants associated with a common source (i.e., road traffic) on mental health. Conversely, [13] find that the significant results of traffic noise on mental health are attenuated when adjusting for other types of pollution (such as PM_2.5_), but that the significant effects of PM_2.5_ on the hazard rate of schizophrenia spectrum disorder, anxiety and depression were only slightly reduced when adjustment was made for the other exposure variables such as traffic noise. Unfortunately, data availability on other exposures is limited for our sample, particularly for historical periods.

Other limitations include the fact that PM_2.5_ annual average concentrations are rounded to the nearest 1 μg/m^3^ (a condition of data access to protect respondents’ anonymity), which reduces the variation in PM_2.5_ concentrations across time and space. In addition, while ambient PM_2.5_ concentrations are most commonly used in studies of this kind, personal exposure is influenced by the different microenvironments or activities an individual experiences (e.g., time in traffic, indoor sources, second-hand tobacco smoke, occupational exposure, and degree of penetration of ambient air pollution into homes, etc.) [28] and is much more difficult to measure. For future work of this kind, it would be particularly useful also to be able to match historical exposures dating back to early childhood to later life mental health outcomes, via complete address histories. As density of ground sensor networks improves (at least in developed countries), more granular exposure estimates should also provide greater sample variation. As in many other applications in the literature, individual-level exposure to PM_2.5_ is calculated using land-use regression models to determine approximate annual concentrations at study participants’ residential addresses. While individuals’ activity patterns also increase exposure misclassification, alternative methods such as using distance from major roads [4] and self-reported measures [29] are problematic, and personal exposure monitoring remains prohibitively expensive for large-scale studies [4].

## Data Availability

Researchers interested in using the TILDA data used in this paper may access the data on request via www.tilda.ie. Statistical analysis was conducted using the statistical software programme Stata 15. Code for this analysis is available on request from the authors.

## Appendix 1

### Data on historical addresses

As part of Wave 3 data collection for The Irish Longitudinal Study on Ageing (TILDA), participants were asked to report the addresses at which they have lived throughout their lives. The residential history module (RHM) was administered as part of the self-completion questionnaire (SCQ) and asked participants the following: “*Where did you previously live? Please start with the most recent previous address first and then the second most recent, and so on*”. The questionnaire provided space for respondents to provide exact address details for up to ten locations where they have resided. A geocoded dataset of the responses to this questionnaire, supplemented with the current recorded addresses of study participants as collected as part of the primary TILDA interview, was provided to the researchers for the present study (*n* = 4,674).

An extensive data cleaning exercise was undertaken to ensure the validity of the residential history information used. The primary goal of the data validation exercise was to create a respondent-year panel dataset detailing the location at which each respondent resided between 1998–2014. An overview of the steps taken to generate this dataset is provided below:

#### Preliminary data cleaning

We first conducted an initial data cleaning exercise in which clear errors in the data were identified and rectified. This exercise included a check for duplication of respondents and the identification of cases where the stated duration of residence was unreasonable (e.g. negative). We also removed geographic coordinates that had been assigned to address locations that did not correspond to a valid location in either the Republic of Ireland or Northern Ireland. While all those included in the baseline interview in 2009/2010 were residents of the Republic of Ireland, those who subsequently moved to Northern Ireland (but not other countries) were followed up for interview.

#### Classify respondents’ engagement with the RHM questionnaire

Since the RHM was contained in the TILDA SCQ, participants were not compelled to complete the questionnaire fully. Consequently, there was significant variation in the extent to which participants engaged with the exercise. Identifying individuals who did not engage at all was essential, as any observed data pertaining to these individuals could not have been derived from the RHM. These data were instead derived from the TILDA current address dataset, which did not require further validation as it was not subject to the same likelihood of reporting errors as the RHM. We thus restricted further data cleaning and imputation steps to individuals identified as giving some information beyond their current address in the RHM.

#### Improve location quality

Not all reported addresses had been assigned an exact geographic location in the version of the RHM data provided to the researchers. We employed several strategies to assign the most detailed geographical identifier possible to incomplete addresses. In many cases, we could identify the county (one of twenty-six in the Republic of Ireland) to which an incomplete address pertained. To assign addresses to the appropriate county, we employed the following strategies:

A. We developed a correspondence table to assign incomplete but known address entries to the appropriate county.
B. Where respondents indicated that they have always lived at the same address or identified an additional period during which they lived at their current address, we assigned the geo-location of their current address to that entry.
C. We performed an automated text search algorithm to the remaining incomplete addresses, allowing us to match some of the remaining entries to the relevant county.

#### Deal with invalid year-of-residence information

In some cases, respondents reported locations where they have lived but did not detail the years during which they resided at these addresses. Such cases were necessarily excluded from our analysis. We also took several actions to resolve cases where it appeared that the years that respondents reported living at an address were incorrect. First, where the order of years was backwards on the RHM form, we assumed this occurred because of a genuine error and reversed the order accordingly. Second, where respondents’ age indicated that a reported address was inhabited before their birth, we adjusted the entries as follows: If the respondent’s sequence of address entries suggested that they ‘moved out’ of the address before their date of birth, we removed the entry entirely. If the sequence of entries indicated that they moved away from the location after birth, we assumed that the location corresponded to the respondent’s address from their approximate year of birth.

#### Geocode validation

The geographic coordinates assigned to each RHM address were not generated as part of the present analysis. We carried out a set of verification exercises to ensure that these coordinates referenced exact residential addresses rather than the centroids of any larger geographic unit. We assigned each set of address coordinates to several official geographic administrative units containing the coordinates and calculated the distance from that address to the unit’s centroid. Specifically, we linked each set of coordinates to its county, electoral district, and small area administrative unit as designated by the Central Statistics Office of Ireland. The exercise revealed no significant concerns that the reported coordinates referred to administrative areas rather than exact addresses.

#### Create a respondent-year panel of address data

We transformed the RHM response dataset into a respondent-year panel of addresses in which we assigned the appropriate address to each year between participants’ estimated year of birth (based on age at the time of interview) and their interview year. Frequently, we observed overlaps in the dataset such that more than one address may be assigned to a given year. We took the following approach to address this issue: First, we created a distance matrix between all the addresses each participant reported over their lives and extracted the straight line distance between conflicting addresses. If that distance was zero, we removed the conflict. For the remaining conflicts, we adopted the practice of taking the first address in the conflict series. This equated to using the address reported first in the RHM questionnaire. The underlying logic was that the information reported first was likely to be the most accurate. We applied one exception to this rule: Where an address within the Republic of Ireland conflicted with an indicator that the respondent was living abroad at the time, we coded the respondent as having been living abroad. A typical migration pattern among this cohort was to have spent a spell living abroad before returning to live in the Republic of Ireland. Since this usually constitutes a significant life event, we assume that respondents will recall it accurately.

#### Combine all valid data to maximise sample size

Many respondents (including those who do not engage with the RHM at all) will have lived at their current address throughout the period of analysis in this paper (1998–2014). As such, we could supplement the information from the RHM with information on respondents’ current addresses as recorded as part of the primary TILDA interview. In the Wave 3 face-to-face interview, participants were asked: *For how many years have you lived at this address?* We used the responses to this question to construct a respondent-year panel, similar to that created from the RHM data, that ran from the first year respondents reported being at that address up to the year of interview. The combined RHM and current address panels (for *n* = 4,497 respondents) are then used for linking with historical air pollution data. See also Fig. 1.

## Appendix 2

### Full model results

**Table 6:** OLS regression results for Z-standardised CES-D depressive symptoms scale using linear rounded long-term average PM_2.5_ concentration

Variables	Model 1				Model 2			
	Coef	CI high	CI low	*P* value	Coef	CI high	CI low	*P* value
PM_2.5_ concentration	0.0325	0.0097	0.0553	0.0051	0.0312	0.0093	0.0531	0.0053
Age					-0.0454	-0.1010	0.0103	0.1100
Age^2^					0.0002	-0.0001	0.0006	0.2290
Female					0.1800	0.1130	0.2480	0.0000
Male					REF			
*Employment status*								
Employed					-0.1070	-0.1960	-0.0188	0.0175
Retired					REF			
Other					0.0795	-0.0269	0.1860	0.1430
*Highest level of education achieved*								
Primary/none					0.0990	0.0056	0.1920	0.0377
Secondary					REF			
Third/higher					0.0157	-0.0575	0.0888	0.6750
Married					-0.2410	-0.3230	-0.1580	0.0000
LT health limitation					0.4810	0.3830	0.5790	0.0000
Alcohol consumption problem					0.2360	0.1270	0.3460	0.0000
Missing alcohol cons					0.0929	-0.0904	0.2760	0.3200
5+ medications					0.1300	0.0385	0.2220	0.0054
Medical card					0.1220	0.0423	0.2010	0.0026
Never smoker					REF			
Past smoker					0.0665	0.0009	0.1320	0.0470
Current smoker					0.2780	0.1410	0.4150	0.0000
Constant	-0.249	-0.425	-0.0731	0.00556	1.491	-0.469	3.451	0.136
Observations	3,407				3,407			
*R* ^2^	0.003				0.110			

**Table 7:** OLS regression results for Z-standardised HADS-A anxiety scale using linear rounded long-term average PM_2.5_ concentration

Variables	Model 3				Model 4			
	Coef	CI high	CI low	*P* value	Coef	CI high	CI low	*P* value
PM_2.5_ concentration	0.0281	0.00523	0.0511	0.0161	0.0380	0.0158	0.0602	0.0008
Age					-0.0429	-0.0974	0.0116	0.1230
Age^2^					0.0001	-0.0002	0.0005	0.5130
Female					0.2470	0.1810	0.3140	0.0000
Male					REF			
*Employment status*								
Employed					-0.0267	-0.1160	0.0623	0.5560
Retired					REF			
Other					0.1110	0.0109	0.2100	0.0297
*Highest level of education achieved*								
Primary/none					0.0864	-0.0054	0.1780	0.0652
Secondary					REF			
Third/higher					0.0769	0.0045	0.1490	0.0374
Married					0.0004	-0.0773	0.0782	0.9910
LT health limitation					0.3320	0.2380	0.4270	0.0000
Alcohol consumption problem					0.3590	0.2480	0.4690	0.0000
Missing alcohol cons					0.3110	0.1210	0.5010	0.0013
5+ medications					0.1160	0.0279	0.2050	0.0099
Medical card					0.1770	0.0934	0.2600	0.0000
Never smoker					REF			
Past smoker					0.0454	-0.0231	0.1140	0.1940
Current smoker					0.1190	-0.0067	0.2440	0.0635
Constant	-0.393	-0.0384	0.0171	1.605	1.5410	-0.3940	3.4750	0.1180
Observations	3,407				3,407			
*R* ^2^	0.002				0.107			

**Table 8:** OLS regression results for Z-standardised CES-D depressive symptoms scale using categorical rounded long-term average PM_2.5_ concentration

Variables	Model 1b				Model 2b			
	Coef	CI high	CI low	*P* value	Coef	CI high	CI low	*P* value
*PM*_*2.5*_ *category*								
5	0.0679	-0.1310	0.2670	0.5030	0.0159	-0.1760	0.2070	0.8710
6	0.0540	-0.0427	0.1510	0.2740	0.0648	-0.0251	0.155	0.1570
7	REF				REF			
8	0.0452	-0.0471	0.1370	0.3370	0.0524	-0.0337	0.1390	0.2330
9	0.0758	-0.0811	0.2330	0.3440	0.1040	-0.0441	0.2510	0.1690
10	0.1210	-0.0160	0.2570	0.0836	0.1540	0.0228	0.2840	0.0214
11	0.2370	0.0920	0.3830	0.0013	0.2000	0.0608	0.3380	0.0048
12	0.3590	-0.1240	0.8420	0.1450	0.2710	-0.2100	0.7530	0.2700
Age					-0.0449	-0.1010	0.0111	0.1160
Age^2^					0.0002	-0.0001	0.0006	0.2390
Female					0.1800	0.1120	0.2470	0.0000
Male					REF			
*Employment status*								
Employed					-0.1070	-0.1960	-0.0189	0.0174
Retired					REF			
Other					0.0801	-0.0262	0.1860	0.1400
*Highest level of education achieved*								
Primary/none					0.0967	0.0031	0.1900	0.0428
Secondary					REF			
Third/higher					0.0145	-0.0586	0.0876	0.6980
Married					-0.2430	-0.3260	-0.1610	0.0000
LT health limitation					0.4790	0.3810	0.5760	0.0000
Alcohol consumption problem					0.2330	0.1230	0.3420	0.0000
Missing alcohol cons					0.0909	-0.0938	0.2760	0.3350
5+ medications					0.1350	0.0431	0.2270	0.0040
Medical card					0.1200	0.0409	0.2000	0.0030
Never smoker					REF			
Past smoker					0.0649	-0.0008	0.1300	0.0527
Current smoker					0.2770	0.1390	0.4150	0.0000
Constant	-0.0576	-0.116	0.000812	0.0533	1.658	-0.302	3.617	0.0973
Observations	3,407				3,407			
*R* ^2^	0.004				0.117			

**Table 9:** OLS regression results for Z-standardised HADS-A anxiety scale using categorical rounded long-term average PM_2.5_ concentration

Variables	Model 3b				Model 4b			
	Coef	CI high	CI low	*P* value	Coef	CI high	CI low	*P* value
*PM*_*2.5*_ *category*								
5	0.0285	-0.185	0.242	0.794	-0.0239	-0.2300	0.1820	0.8200
6	0.149	0.0528	0.245	0.00239	0.1210	0.0284	0.2130	0.0104
7	REF				REF			
8	0.154	0.0641	0.245	0.000818	0.1400	0.0553	0.2260	0.0012
9	0.211	0.0464	0.375	0.0119	0.2100	0.0539	0.3670	0.0084
10	0.149	0.0120	0.286	0.0331	0.2070	0.0758	0.3390	0.0020
11	0.202	0.0568	0.347	0.00643	0.2070	0.0690	0.3450	0.0033
12	0.422	-0.0758	0.920	0.0965	0.4080	-0.0548	0.8710	0.0840
Age					-0.0437	-0.0983	0.0109	0.1170
Age^2^					0.0001	-0.0002	0.0005	0.4880
Female					0.2490	0.1820	0.3160	0.0000
Male					REF			
*Employment status*								
Employed					-0.0258	-0.1150	0.0630	0.5690
Retired					REF			
Other					0.1090	0.0091	0.2080	0.0325
*Highest level of education achieved*								
Primary/none					0.0844	-0.0074	0.1760	0.0715
Secondary					REF			
Third/higher					0.0774	0.0052	0.1500	0.0357
Married					-0.0028	-0.0806	0.0750	0.9440
LT health limitation					0.3290	0.2350	0.4240	0.0000
Alcohol consumption problem					0.3530	0.2420	0.4640	0.0000
Missing alcohol cons					0.3040	0.1150	0.4930	0.0016
5+ medications					0.1240	0.0355	0.2130	0.0061
Medical card					0.1790	0.0958	0.2620	0.0000
Never smoker					REF			
Past smoker					0.0449	-0.0237	0.1130	0.1990
Current smoker					0.1240	-0.0022	0.2490	0.0542
Constant	-0.110	-0.169	-0.0514	0.000248	1.747	-0.192	3.686	0.0775
Observations	3,407				3,407			
*R* ^2^	0.007				0.111			
